# A reply to ‘Alteration of steroidogenesis in boys with autism spectrum disorders’

**DOI:** 10.1038/s41398-021-01393-9

**Published:** 2021-05-10

**Authors:** Benedikt Andreas Gasser, Johann Kurz, Bernhard Dick, Markus Georg Mohaupt

**Affiliations:** 1 DSBG, University of Basel, 4052 Basel, Switzerland; 2 Intersci Research Association, Karl Morre Gasse 10, 8430 Leibnitz, Austria; 3 Department of Clinical Research, University of Bern, 3010 Berne, Switzerland; 4 Teaching Hospital Internal Medicine, Lindenhofgruppe, 3006 Berne, Switzerland

**Keywords:** Scientific community, Autism spectrum disorders

Dear Editor,

Janšáková et al. published a study in *Translational Psychiatry* that indicated that steroidogenesis in boys (average age 4.4 ± 1.1 years) with autism spectrum disorders differed from that in healthy controls^1^. The study involved comprehensive GC-MS analyses of blood samples from boys with autism and showed alterations of steroid hormones, especially higher androgen levels before puberty in boys with autism than in healthy controls. Janšáková et al. referred to our work in contrast to their findings in the discussion section and mentioned higher concentrations of unconjugated steroids, including most measured androstanes such as androsterone, etiocholanolone, androstenediol, 11β-hydroxyandrosterone, 11β-hydroxyetiocholanolone, dehydroepiandosterone, 5-androstene-3β and 17β-diol, in the urine during puberty in boys with autism than in controls^2^. This fact was explained by Janšáková et al. by precocious adrenarche leading to premature puberty, which has been described even in individuals with autism^3^^,4^. In summary, lower sulfated androstane levels were reported by Janšáková et al. in boys (average age 4.4 years) with autism before puberty than in our sample of boys (average age 15.3 ± 2.9 years for Asperger and 13.6 ± 3.6 years for Kanner’s syndrome) with autism during puberty. Thus, the relation to the onset of puberty may be a key factor explaining the different results. Indeed, we believe that differences in the findings result from the lower average age and, consequently, pubertal status. In fact, human steroidogenesis is strongly age dependent, particularly in children and pubescents, supporting the relevance on this argument. However, additional factors might contribute to differences in the findings such as (i) measurement of steroid hormones in plasma versus urine, (ii) the backdoor pathway of androgen synthesis and (iii) altered activity of enzymatic pathways.

## Steroid hormone measurement in plasma versus urine

Janšáková et al. performed measurements from plasma in contrast to our sample with urine analysis. We believe that the UGT polymorphism is especially relevant^5–9^. We mainly measured products of phase II metabolism in our urine samples. Consequently, it is important to understand that endocrine disruptors, UGT polymorphisms, drugs, alcohol and dietary supplements may affect the glucuronidation of steroid hormones, cofounding the results and consequently complicating direct comparisons^5–9^. We were also able to identify increased levels of androgens, such as testosterone, androstenediol, 11β-hydroxyandrosterone and the mineralocorticoid tetrahydroaldosterone, in the urine of pubertal autistic girls. Although the results were less pronounced than in boys, this is in line with the statement of Janšáková et al. that there is involvement of all three layers of the adrenal gland (zona glomerulosa, zona fasciculata and zona reticularis) and highlights the relevance of this organ^10^.

## Backdoor pathway of androgen synthesis

When focusing on biochemical pathways of androgen synthesis, two major pathways should be considered: the classic and backdoor pathways^11–14^. In short, human steroidogenic enzymes efficiently catalyse all the required steps in a route to dihydrotestosterone that does not involve the testosterone intermediate, called the backdoor pathway of androgen synthesis^15^. The backdoor pathway of androgen synthesis provides a faster route to dihydrotestosterone^16^. It has been reported that backdoor pathways play an important role in human hyperandrogenic disorders such as 21-hydroxylase deficiency, Fragile-X-syndrome or altered sexual development^17–22^ but also in physiological states such as mini puberty (a short increase in androgen levels in the first year of life)^15^^,^^21–23^. O’Shaugnessy et al. showed that the main androgen of the backdoor pathway is androsterone^22^. In contrast, eticholanolone is mainly present in the classic pathway of androgen synthesis, which means that increased ratios of androsterone-to-eticholanolone may explain the potential activity of the backdoor pathway^15^^,23–25^. Consequently, androsterone-to-eticholanolone ratios are used as indicators for backdoor pathway activity in androgen synthesis, and alterations are associated with autism^15–21^^,26–28^. Thus, an increase in androsterone-to-eticholanolone ratios should be indicative of increased activity of this pathway^22^^,^^28^. However, in our sample, in contrast to the analyses of Janšáková et al., the metabolites androsterone and eticholanolone showed approximately twofold higher concentrations than those in controls, and as a consequence, the androsterone-to-eticholanolone ratios were not increased^22^^,28^. Based on these analyses, there is no evidence for higher or lower backdoor pathway activity in androgen synthesis^28^. These results are in contrast to those of Janšáková et al., suggesting lower activity of the backdoor pathway, as both metabolites were present at lower levels in boys with autism. Therefore, there is still uncertainty concerning the involvement of the backdoor pathway of androgen synthesis in girls and boys with autism, and explanations remain speculative, particularly because we have data from only relatively small samples.

## Alterations of enzymatic pathways

Enzymatic pathways were differentially altered in the two samples analysed. For example, a 21-hydroxylase deficiency is not clearly detectable in our analyses, yet alterations in 17-hydroxylase, 11-hydroxylase and 11-β hydroxysteroid dehydrogenase were present in our sample. When focusing on 11-β hydroxysteroid dehydrogenase activity, interestingly, cortisol and cortisone metabolites were almost similarly changed in both samples, implying reduced 11-β hydroxysteroid dehydrogenase activity, which is in line with the previously reported role of hypercortisolism in an original description of autism^29^^,30^. In conclusion, analyses of steroid hormones by Janšáková et al., by our group and by others imply that there are higher concentrations of androgens in boys and girls with autism^1–3^^,10^^,31–33^. One main factor is the condition of the adrenal gland before and after puberty. One general premise might be that the zona reticularis is mainly active after puberty^34^. This would explain the higher activity of the CYP17A hydroxylase step in the absence of the lyase step in young children^1^^,34^. Once the zona reticularis of the adrenal gland is activated, increased production of adrenal androgens can be detected through increased lyase activity^1^^,34^. This premise would be supported by findings indicating that even children at the age of 6 still have negligible activity of the adrenal zona reticularis^34^. This premise would be in line with the general finding of higher androgens in boys and girls with autism after puberty^1^^,2^^,12^. It is tempting to speculate about the role of the gonads, and the focus should be on a dysregulated adrenal gland in autism^2^^,12^. Furthermore, it seems that more than a hormonal change underlies the pathology of autism^1^. Altered hormonal profiles might be a result of altered gene expression of hormone-associated or -related genes^1^^,35–37^. For this reason, the steroid hormonal profile and genetic background should be further investigated^1^^,35–37^.

In summary, despite notable differences between studies (measurements in urine in our study versus in plasma in the study by Janšáková et al., as well as different ages of the samples), higher levels of steroid hormones are very likely to suggest in autism. Therefore, the cholesterol hypothesis of autism of Gillberg et al. is in line with the findings of the two studies^38^. However, an encompassing view of all steroid hormone interactions is difficult. It seems that steroid hormone cascades act as complex networks with multiple entries and parallel signalling pathways in which hormones affect each other and are regulated by feedback and feedforward mechanisms^38^. Furthermore, we do not know all players in these steroid networks, and these networks are not hierarchically or logically structured. The apparent complexity makes intuitive or mathematical prognosis impossible at the moment, easy applicable diagnostic markers are out of view^39^ and further research is warranted to deepen our understanding of alterations in steroid hormones in children with autism.
